# India-US collaboration to prevent adolescent HIV infection: the feasibility of a family-based HIV-prevention intervention for rural Indian youth

**DOI:** 10.1186/1758-2652-12-35

**Published:** 2009-11-19

**Authors:** Asha Banu Soletti, Vincent Guilamo-Ramos, Denise Burnette, Shilpi Sharma, Alida Bouris

**Affiliations:** 1 School of Social Work, Tata Institute of Social Sciences, Mumbai, India; 2 Columbia University School of Social Work, New York, NY, USA; 3 School of Social Service Administration, University of Chicago, USA

## Abstract

**Background:**

Despite the centrality of family in Indian society, relatively little is known about family-based communication concerning sexual behaviour and HIV/AIDS in rural Indian families. To date, very few family-based adolescent HIV-prevention interventions have been developed for rural Indian youth. This study conducted formative research with youth aged 14 to18 years and their parents in order to assess the feasibility of conducting a family-based HIV-prevention intervention for rural Indian adolescents.

**Methods:**

Eight focus groups were conducted (n = 46) with mothers, fathers, adolescent females and adolescent males (two focus groups were held for each of the four groups). All focus groups consisted of same-gender participants. Adolescents aged 14 to18 years old and their parents were recruited from a tribal community in rural Maharashtra, India. Focus group transcripts were content analyzed to identify themes related to family perceptions about HIV/AIDS and participation in a family-based intervention to reduce adolescent vulnerability to HIV infection.

**Results:**

Six primary thematic areas were identified: (1) family knowledge about HIV/AIDS; (2) family perceptions about adolescent vulnerability to HIV infection; (3) feasibility of a family-based programme to prevent adolescent HIV infection; (4) barriers to participation; (5) recruitment and retention strategies; and (6) preferred content for an adolescent HIV prevention intervention.

**Conclusion:**

Despite suggestions that family-based approaches to preventing adolescent HIV infection may be culturally inappropriate, our results suggest that a family-based intervention to prevent adolescent HIV infection is feasible if it: (1) provides families with comprehensive HIV prevention strategies and knowledge; (2) addresses barriers to participation; (3) is adolescent friendly, flexible and convenient; and (4) is developmentally and culturally appropriate for rural Indian families.

## Background

Preventing the transmission of HIV in India remains a significant goal for global public health. In 2007, an estimated 2.4 million Indians were living with HIV [1]. Among the many states that comprise India, the western state of Maharashtra bears one of the highest HIV burdens. At least 20% of India's estimated HIV cases are in Maharashtra, and the state has an overall prevalence rate of 0.74% [2]. Although adolescents and young adults aged 15 to 29 years old account for approximately 25% of India's total population, they represent 31% of the country's AIDS cases, indicating that many Indians are becoming infected during adolescence or early adulthood [2,3].

Recognizing that the successful prevention and treatment of HIV/AIDS requires international cooperation across multiple disciplines, the Indian Minister of Health and Family Welfare and the US Secretary of Health and Human Services signed a bilateral agreement in 2006 to collaborate on the prevention of sexually transmitted infections (STIs) and HIV/AIDS in India [4,5]. The overall goal of the bilateral agreement is to "promote and develop cooperation in the fields of HIV/AIDS and STI prevention, research, treatment and care, infrastructure development, training, and capacity-building on the basis of reciprocity and mutual benefit" [5]. The bilateral agreement also identifies a number of key areas for cooperation between India and the US, including "developing innovative intervention strategies for the prevention and treatment of HIV/AIDS" [5].

Our study is a collaboration between social scientists in India and the United States that was conducted as part of the Indo-US bilateral agreement. The overall goal of the collaboration is to conduct formative research that will inform the development of a family-based intervention to prevent HIV infection among Indian youth living in a rural community in Maharashtra. The family-based intervention will integrate the principles of "highly-active HIV prevention" by incorporating both biomedical (e.g., condoms) and behavioural prevention strategies that have been deemed efficacious for preventing HIV transmission [6].

A secondary goal is to scale up the knowledge base and research capacities of both Indian and American social scientists to develop and implement innovative, culturally appropriate, effective and sustainable HIV/AIDS prevention and treatment programmes. The results of this study represent the first of several formative research projects in support of these two goals.

The overall objective was to gain insight into diverse family perspectives on the feasibility and acceptability of a family-based adolescent HIV prevention programme for rural Indian families. The proposed intervention is distinct from previous prevention approaches in that parents will be targeted as agents of change who can provide their adolescents with the guidance, information and strategies necessary to reduce their risk of HIV infection.

To date, we know of no family-based adolescent HIV-prevention programmes for rural Indian youth. The majority of adolescent prevention programmes have tended to target adolescents via peer models or school-based programmes [7-9], or have focused predominantly on urban areas. As a result, relatively little is known about the familial and contextual factors that might promote or hinder the success of a family-based HIV prevention intervention for rural youth.

This study focused on adolescents aged 14 to 18 years old and their families who reside in a rural community near Mumbai and Pune in Maharashtra. Rural adolescents in Maharashtra were targeted for several reasons. First, Maharashtra continues to bear a disproportionately high burden of HIV cases in India [2]. In addition, research with rural youth in Maharashtra suggests that HIV knowledge is low. For example, in a study with rural Maharashtran girls and women aged 15 to 24 years old, only 49% indicated that they were aware of AIDS and only 60% reported that AIDS could be avoided [10].

Sexual behaviour remains the leading cause of HIV infection in India [11], and complex factors underlie rural youth's vulnerability to HIV. In Maharashtra, many rural young men migrate to cities, particularly Mumbai, in search of economic opportunities. While they are in urban areas, young men may have sexual relationships with women, including sex workers [12]. When male migrants return to their rural homes to marry and begin families, this migration creates a bridge for HIV infection. In addition, studies have also documented high rates of unprotected anal intercourse among rural men who have sex with men [13].

Although male adolescents report higher rates of sexual activity than females, female adolescents are also vulnerable to HIV. A complex combination of factors related to increased biological susceptibility, low levels of education, poverty and gender inequality heighten vulnerability for many females [8]. Many young women in Maharashtra do not complete secondary school. Some young women enter early marriages or commercial sex work, and gender inequality creates power differences that create formidable barriers to consistent condom use. Among young people aged 15 to 24 years, the number of women with HIV/AIDS is estimated to be almost twice that of young men [14]. Taken together, these factors suggest that rural adolescents are a vulnerable group of young people.

A growing body of research conducted with young people in developing contexts indicates that parents can influence the sexual decision making of their adolescent children [15-17]. These findings are consistent with the large body of literature from the US, which has found that parents can influence an adolescent's sexual debut [18], condom use [19] and acquisition of STIs [20]. Additionally, a number of parent-based interventions evaluated in the US show that parents can reduce adolescent sexual risk behaviour when given appropriate information and parenting strategies [21-23].

Despite widespread support for the influence of parents on adolescent sexual behaviour, parent-based approaches to preventing adolescent HIV infection in India are rare. Indian culture is often characterized as having strong norms against open discussions of sexual behaviour [24], and Indian families are said to engage in indirect communication about sex [25]. At the same time, many Indian parents are concerned about their children becoming infected with HIV [26,27] and want to help their children make appropriate decisions regarding marriage [27,28].

Research also indicates that Indian adolescents are influenced by their parents. For example, a study in Uttaranchal observed that many young men attributed premarital sex to low levels of parental control and supervision [26]. In addition, a recent study with youth in Pune found that young people were more likely to talk with their parents about romantic relationships than they were with their peers [28]. Moreover, females who reported high levels of parental closeness were less likely to form romantic relationships [28].

Our study is distinct from previous research in several ways. First, it focused on families and parent-adolescent communication about HIV/AIDS as a means of preventing sexual risk behaviour and reducing adolescent vulnerability to HIV. Although the family has been the focus of interventions to help Indian persons living with HIV/AIDS, less research has focused on the family as a way to reduce adolescent vulnerability to HIV/AIDS. Open discussions about sexual behaviour are perceived as taboo in Indian culture [8,24,29]. As a result, relatively little is known about family communication about HIV/AIDS and how best to design a family-based intervention to prevent adolescent HIV infection.

We conducted exploratory research with families to generate insight into an understudied topic in the HIV/AIDS prevention literature. Previous research has tended to interview individual family members, i.e., adolescents [8,10]. In contrast, we conducted focus groups with mothers, fathers, and adolescent males and females in order to obtain a more comprehensive understanding of family perspectives on preventing adolescent HIV infection. In addition, interviewing multiple family members provided insight into possible biases in perceptions versus actual behaviour with respect to parent-adolescent communication about HIV/AIDS.

Finally, a strength of the study is the collaboration and integration of Indian and US perspectives into the development of study protocols and a family-based intervention to prevent adolescent HIV infection.

## Methods

Focus group methodology was selected for several reasons. First, focus groups are ideal for understanding the norms and values of culturally diverse populations [30,31]. In India, focus groups have been used to explore a range of HIV-related issues, including factors that may impact on participation in future HIV vaccine trials [32], on acceptability of a vaginal gel among HIV-negative women [33], and on domestic violence on women's HIV risk [34]. In addition, given the dearth of research on family-based interventions to prevent adolescent HIV infection, focus groups were identified as an ideal methodology to explore the topic with families.

### Community background

The study was conducted in Aghai, a village in the Thane district of Maharashtra. Thane, which is north-east of Mumbai and adjacent to Pune, has a population of 8.1 million, of which 30% is rural. In 1986, the School of Social Work at the Tata Institute of Social Sciences established an Integrated Rural Health and Development Project (IRHDP) in Aghai and its 20 surrounding *padas*, or hamlets. The objectives of the IRHDP are to promote health and education and to effectively utilize and generate local resources for villagers in collaboration with the local primary health centre.

The IRHDP has developed strong community relationships with the local *padas*. As part of its work, the IRHDP also creates a map of each village and keeps records on the nature of health work conducted in each village. Using the IRHDP village social map and the most recent community census, we selected a *pada *with which local health workers had a strong existing relationship, but no special history of HIV/AIDS-related work. In total, there were 41 households in the selected *pada*. Of the 41 households, 25 included at least one unmarried adolescent aged 14 to18 years.

### Recruitment and consent

After the sampling frame was finalized, recruitment was conducted via face-to-face outreach by trained, indigenous recruiters who visited homes with eligible adolescents and invited them and their eligible family members to participate. One target adolescent and one target parent from each family were asked to participate. In cases of two or more eligible adolescents, recruiters invited the youngest to participate.

The target parent and adolescent were asked to join a focus group study that sought to understand family members' perspectives about participating in a family-based programme to help adolescents avoid HIV. As part of the consenting process, families were given basic information related to HIV. Recruiters explained the purpose of the study, the nature of the focus group process, and the right to refuse with no penalty.

A total of 48 individuals were approached to participate in the study and 46 (96%) consented to participate in the study and completed the focus groups. Adolescents received 100 Indian rupees for participating and each parent received 250 Indian rupees (about US$2 and $5, respectively). Institutional Review Board Approval was obtained from both the Tata Institute of Social Sciences (IEC/IRB No: 03/2009) and Columbia University (IRB-AAAC8244); all research protocols complied with the Helsinki Declaration.

### Data collection

Separate groups with mothers, fathers, adolescent females and adolescent males were conducted for several reasons. First, Vissandjée, Abdool, and Dupéré [35] suggest that smaller groups of six to eight participants are ideal for exploring sensitive topics. In addition, triangulating the perspectives of different groups can enhance topic understanding, while homogeneity of group members' experiences can reduce power differentials and promote participant comfort [36,37]. Finally, gender and age are especially salient factors in some non-Western cultures, where younger persons are discouraged from differing with older or more influential persons, or where females may tend to defer to males [38]. Given these factors, the number of participants per group was kept to six or less.

The standard protocol is to conduct at least three focus groups with each type of participant [36,39]. However, the relatively small size of the population in the village and the high degree of homogeneity of families within and across *padas *meant that two groups each with adolescent boys, adolescent girls, mothers and fathers were sufficient to cover the research questions. On average, each group lasted for 1.5 hours.

Focus group venues need to be acceptable, private, convenient, and easily accessible for all participants [35,40]. As the *pada *lacked a common space, the girls and the mothers groups met in the house of the *pada *worker, and the boys and fathers groups met in the house of the *anganwadi *(primary school) teacher. The venues were carefully selected spaces that were well known and respected by community members as this was deemed important to engendering participant trust and comfort in the focus group process by the indigenous research staff. Utmost care was taken to ensure privacy during the focus groups. The presence of onlookers and other distractions were minimized by holding the meetings indoors [41,42], and only the focus group facilitators and consented participants were present at each focus group.

Successful focus group implementation depends heavily on the ability of facilitators to moderate the focus group. In this study, the focus group facilitators consisted of the first and fourth authors, and a team of indigenous data collectors. Although all facilitators were familiar with the cultural and demographic profile of the target population, none resided in the target community. The facilitators led each focus group using a protocol developed by the first three authors, and refined with indigenous project staff and community members.

Facilitators then used a "funnel" approach to frame the development of the questioning route [39,43], which allowed for a wider perspective of individual experiences in the initial stages, followed by specific questioning in subsequent stages to directly answer the research questions. This question route enhanced the consistency of data obtained between groups and assisted in efficient, high-quality data analysis [44].

The questions elicited perspectives about the development and implementation of a family-based community intervention for HIV/AIDS in three core domains: (1) perceptions about and preferred format for planned intervention; (2) preferred methods for implementation; and (3) factors that could potentially foster or inhibit full engagement and participation in the intervention. The same sets of questions were asked in each focus group.

### Data analysis

Each focus group was tape recorded on an audio cassette and a written verbatim transcript was produced in Marathi. The transcript was translated into English and checked for accuracy using a forward-backward translation method [45]. In addition, the translators reviewed the transcripts to ensure conceptual as well as linguistic equivalence in the translation process [46]. In order to minimize potential bias in data analysis and interpretation, we followed Krueger and Casey's [36] guidelines to ensure the analysis process was systematic, sequential, verifiable and continuous.

Four independent coders conducted a content analysis to identify "thematic units", which were defined as frequently occurring sets of explanatory statements [47]. In addition, data were explored for negative incidents and divergent themes [48,49], which added rigour and validity to the results [50,51]. Interrater reliability among the four coders was determined via a frequency count strategy described by Miles and Huberman [49].

Upon completion of coding, each coder independently calculated the frequency that each category and sub-category occurred within the data. The four coders then compared the correspondence in the data analysis. When disagreement occurred, the disagreement was recorded and settled via discussion between the four coders. The total number of agreements was then divided by the total number of agreements and disagreements [49], leading to an interrater reliability of 91%.

## Results

Six primary areas were identified: (1) family-based knowledge about comprehensive HIV prevention strategies; (2) family perceptions about adolescent vulnerability to HIV/AIDS; (3) feasibility of a comprehensive family-based programme to prevent adolescent HIV infection; (4) barriers to participation; (5) recruitment and retention strategies; and (6) preferred content for an adolescent HIV-prevention intervention.

### Family knowledge about HIV/AIDS

There was wide variation in knowledge about HIV/AIDS among adolescents and parents. While most of the adolescent boys and girls reported that they had heard of "AIDS", factual knowledge about HIV/AIDS was varied. For example, while some adolescent boys recognized that AIDS was a "big disease", they did not know what it meant. One youth stated, "I have heard about it but don't know anything about it." In addition, some boys reported incorrect knowledge, such as believing that AIDS caused malaria. In contrast to the lack of accurate knowledge evidenced by some male adolescents, other boys reported detailed information about HIV transmission and its impact on health. One boy stated, "AIDS happens due to the HIV virus."

Of the boys who had some knowledge about HIV/AIDS, they identified a number of possible routes of transmission, including: (1) sexual behaviour between adults or between youth; (2) having multiple sexual partners; (3) being exposed to infected blood; (4) from a pregnant mother to her child; and (5) from exposure to syringes. This group of youth also knew that HIV/AIDS could be treated with medicines, but could not be cured. When asked to identify sources of information about HIV, adolescent boys indicated that they obtained most of their knowledge from the television. Without exception, all of the boys in the focus groups indicated that their parents had not spoken to them about HIV/AIDS.

A similar pattern of results emerged from the focus groups with adolescent girls. For the most part, adolescent girls reported that they heard of the word "AIDS" and were able to identify that it was a disease. While a small number of girls indicated that their knowledge about HIV/AIDS was limited, many were able to identify potential routes of transmission. The most frequently cited mechanisms of HIV transmission included sexual behaviour between men and women, (e.g., "AIDS happens due to sexual contact. AIDS can happen due to a girl-boy or man-women physical relationship"), and through exposure to "infected blood" or a syringe that had been used on an HIV-positive person (e.g., "AIDS can happen if a needle used on an infected person is reused on another person").

Whereas boys identified television as a primary source of information, girls reported learning about HIV/AIDS through the television, newspapers and posters placed at local health centres. In addition, some of the adolescent girls indicated that their teachers in school had discussed HIV/AIDS with them. Like their male peers, adolescent females indicated that their parents had not addressed the topic of HIV/AIDS with them.

Mothers and fathers also reported similar variation in knowledge about HIV/AIDS. While some parents reported very detailed information about HIV and how it could be transmitted, others indicated that they knew very little. In the mother focus groups, one mother explained her knowledge about HIV/AIDS as:

It [AIDS] can happen to anyone. From small children to anybody. It can happen to anybody who gets pricked by an infected needle. When in mother's womb ... it can happen then too. If she comes to know about it, then she can take medicines and save her child from the disease. Only she can't breast feed. This much I know.

This same level of detail was evidenced in the father focus groups, where one father explained how he arrived at his knowledge about HIV transmission:

Yes I know [about AIDS], the doctor gives information. Or the information is on the board (at the health centre). I know how to read so I was able to read. It is written that "Don't go to outside women, because if she has AIDS then it can happen to us." When we go to the doctor and get an injection, if it is not sterilized then we can get it. We go to the barber and if an old blade is used and if there is blood on it and if we get wounded from that blade then we too can get AIDS.

Of the parents who were aware of HIV, parents discussed sexual behaviour between men and women, sexual behaviour with female sex workers (e.g., with "outside women"), infected syringes, "contaminated blood", and mother to child transmission as possible routes of HIV transmission. In addition, this group of parents was also aware that HIV/AIDS could be treated with medication.

In contrast, other parents indicated that they knew very little about HIV/AIDS. In both the mother and father focus groups, a small number of parents admitted to knowing "nothing" about HIV/AIDS, how the virus was transmitted, or such methods as condoms for reducing one's risk. For example, one mother stated, "No [I] didn't know [about AIDS] before [the focus group], now that you are telling, that we are hearing."

This was echoed in the father focus groups, where one father stated that HIV could be transmitted by sharing drinking water with an HIV-positive person. Still other parents were unaware that HIV could be prevented within the family, as evidenced by a father's statement that, "If one woman gets it [AIDS], one man gets it, and then everyone in the family gets it." When asked to identify their primary sources of information about HIV/AIDS, the majority of mothers discussed learning about HIV/AIDS from the television while fathers indicated that they had received information via the radio, television, doctors, the health centre and written materials.

Largely missing from the focus group discussions was mention of the role of correct and consistent condom use as a means of protecting oneself from HIV. Neither parents nor adolescents discussed condoms as an optimal strategy for protecting oneself from HIV. Families reported low levels of knowledge related to correct and consistent condom use. In general, focus group participants provided less clear feedback in relation to the use of condoms.

Most of the families were uncomfortable with their adolescent children being sexually active outside of marriage. However, in those instances where parents knew that adolescent sexual behavior was occurring, parents reported having great concern in keeping their children safe from potential health consequences associated with risky sexual activity. For instance, one father stated that he observed his adolescent son and some of his son's friends going into a brothel in a city located in close proximity to the target community. The brothel is a known establishment for sex work. The participating father expressed disapproval of his son's seeking out sex workers. However, he also reported wanting his adolescent son to protect himself from sexually transmitted diseases by using condoms if he was to continue frequenting this establishment.

### Family perceptions about adolescents' vulnerability to HIV/AIDS

The second theme that emerged from the focus groups focused on the extent to which families perceived that adolescents were vulnerable to HIV/AIDS. In general, adolescents did not believe that HIV/AIDS was something that directly affected them. Although a small number of boys indicated that HIV/AIDS could occur outside of urban areas, the majority believed that HIV/AIDS occurred mostly in cities.

One boy explained how there are "bad" boys in the city and "good" boys in the village. This feeling was summarized by one male adolescent who said that he felt there was limited possibility of HIV spreading in the local community. In both the male and female focus groups, youth reported that they did not know anyone who was living with HIV/AIDS.

Like their adolescent children, mothers did not readily identify knowing anyone with HIV/AIDS. Although several mothers stated that HIV/AIDS could affect "anyone", another stated, "Where it [HIV/AIDS] is where it is not, we do not have any idea." In addition, mothers echoed the sentiments of their adolescent children about who became infected with HIV/AIDS. One mother said, "One who goes 'wrong' will get the disease."

In contrast to the mother and adolescent focus groups, a number of fathers spoke about their personal experiences knowing people affected by HIV/AIDS. One father shared the story of a friend who had contracted HIV via a sexual relationship with a woman:

There was someone I knew who visited another women and he started getting fever regularly. Later on we came to know that he has AIDS and he died. I know this because this happened in front of us.

Still another shared the story of a friend who had travelled from the village to Mumbai:

There was a friend of mine, he used to roam around, used to go to Mumbai. He must have been doing such things there so he got AIDS. Later, doctor told that he had got AIDS. After that, for some time he tried, but later he passed away.

Finally, another father shared his familiarity with HIV/AIDS via his work as a truck driver, "I am a driver and these things [AIDS] happen earlier to us."

Unlike their adolescent children, both mothers and fathers believed that their children were at risk for HIV. Perceptions of adolescent vulnerability were most often discussed in the context of economic constraints that forced children to seek work in neighbouring villages or cities.

Mothers recognized that they could not effectively monitor their children's whereabouts when they left home for work and believed this opened the door for sexual behaviour that could expose their children to HIV. Fathers, who had also discussed their own experiences migrating for work or knowing other adults who had migrated for work, believed that travelling to other villages and cities for economic opportunities placed their children at risk for HIV, "They are outside and they feel it is a need so they have sexual relationships." one father said.

### Feasibility of a family-based programme

All four groups of stakeholders indicated that a family-based intervention was a feasible and culturally acceptable way to prevent HIV transmission among adolescents. For example, both adolescent males and females indicated that they were interested in participating in a family-based intervention that would provide them with comprehensive skills and information to reduce their risk of acquiring HIV. When asked to elaborate, adolescent males indicated that they listened to their parents and respected their beliefs and opinions more than they would an "outsider".

Related to this, adolescent males also recognized that a comprehensive family-based approach could be easily integrated into their daily life. As one adolescent male stated, "It is beneficial if information and skill are given by families because someone who comes from outside will only be there for one day but if you err then family is there every day to tell."

Similarly, adolescent girls believed it would be beneficial to have their parents talk to them about HIV/AIDS and that their parents could be a good source of knowledge and skills. Family-based approaches were praised by girls for their inclusiveness. As one girl said, "We don't feel that anybody should be excluded like girls, boys, mothers, fathers. All should come together for the programme."

In addition, adolescent girls believed that their parents could be effective teachers, especially if given correct information and skills about HIV/AIDS.

Mothers and fathers were open to participating in a family-based programme and believed that a comprehensive family-based programme was feasible. All of the parents were concerned about their child's health and wellbeing, and many were aware that HIV/AIDS posed a serious health risk. Like their adolescent children, parents recognized that a family-based approach might be more successful than other types of programmes. As one father stated:

Parents will say and children will listen, but when an outsider comes and talks then there are many things that children will feel shy to speak to you as an outsider, they will not talk the way we are talking to you ... they will feel shy. That's why it is important for parents to explain to them.

Without exception, parents wanted to talk with their children about HIV/AIDS. As one mother stated, "It is the duty of parents to speak to their daughters and sons about these issues. We should only make them understand and if we don't tell them how will they know?"

At the same time, only a small number of parents said that they had actually talked with their children about topics like HIV/AIDS and sexual behaviour. Overall, both mothers and fathers felt that they lacked the necessary information and skills to communicate effectively with their children. In particular, parents felt they lacked adequate information related to correct and consistent condom use, and would need additional help if they were to instruct their teens on this topic. For their part, mothers wanted factual information and believed that their children would listen to them if given proper information. One mother said, "You should teach us. What all we don't know, you must tell us. You should give information to parents as well as children. Then even we will be able to speak."

Similarly, fathers believed that they should speak with their children about sexual behaviour and HIV/AIDS, but needed additional support to have effective conversations. Fathers believed that a family-based HIV prevention programme would be especially useful as it could "give us advice which we can give our children".

### Barriers to participating in a family-based intervention

Adolescents and parents identified a number of barriers to participating in a programme. Identified barriers focused on three primary areas: (1) embarrassment and fear of discussing sensitive topics like sexual behavior, correct and consistent condom use and HIV/AIDS, especially when considering gender dynamics in Indian families; (2) stigma surrounding HIV/AIDS; and (3) economic and environmental constraints.

Both adolescents and parents discussed the need to address potential feelings of embarrassment. For adolescents, feelings of discomfort emerged around the idea of having a mixed-gender programme. Although some adolescent boys and girls felt comfortable with a mixed-gender HIV/AIDS intervention, the majority wanted separate groups and felt that family communication might be more effective between mothers and daughters and between fathers and sons. The discussion of same-gender communication in the family system was more often discussed by girls than by boys. If a programme was going to use a mixed-gender approach, adolescent girls recommended involving the entire community, e.g., individuals, households, families, schools and villages, as this would lessen their embarrassment.

For their part, parents discussed how fear of negative consequences could deter their participation in a family-based programme. In the mother focus groups, some women indicated that although they wanted to talk about HIV/AIDS with their children, they were worried that their adolescents would react negatively to such conversations. However, mothers were unable to provide specific examples of how youth might respond in a negative way. Unlike their children, mothers did not identify gender in the family system as a potential barrier to participation.

In contrast, fathers indicated that they might be embarrassed discussing a sensitive topic like sexual behaviour or HIV/AIDS with their adolescent daughters. As one father stated:

When our daughters have come to age (meaning has become a teenager), it becomes awkward to speak with her by a father. So one can ask the mother of the girl to speak to her. Mother-daughter communication happens.

This sentiment was echoed by other fathers, who suggested that embarrassment could be overcome by supporting "mother-daughter" and "father-son" communication. At the same time, other fathers felt that a family-based programme was not embarrassing. "It sometimes gets a little awkward for the parents to speak to their children, but we don't feel that," one father said.

In addition to potential feelings of embarrassment, another barrier to participation addressed the role of stigma related to HIV/AIDS. Adolescents, mothers and fathers all described stigma related to HIV/AIDS. In the adolescent male focus groups, some boys indicated they would feel shy or scared about discussing the topic of HIV. For example, one boy stated, "This is a bad disease, and it feels weird so even I don't speak."

Moreover, boys discussed the fear and stigma towards people living with AIDS and how people in the village responded. One boy said, "If someone amongst us has AIDS then people will try to stay away from him. People might criticize or make fun of him or might tell him something." Another boy said, "Anything can happen to such a person so he is kept outside the house in the village."

Girls expressed similar fears about people living with HIV/AIDS, as evidence by the statements, "Nobody will even speak to him [person living with HIV/AIDS]" and "People will stay away from him [person living with HIV/AIDS] ... because we will get the disease."

Similarly, mothers also indicated that individuals who were known to be HIV positive were shunned by the rest of the community. One mother stated, "If someone comes to know [about having AIDS] then who will go to his house, nobody will eat from his house not even drink water." Fathers also discussed the role of stigma towards people living with HIV/AIDS and believed that it could deter some people from participating, as is clear from this statement, "This programme is on AIDS so people will not come ..."

At the same time, fathers also believed that stigma surrounding HIV/AIDS could be overcome by discussing the importance of prevention with community members and by highlighting the benefits for adolescents and future generations.

The final barrier to participation focused on the role of economic and environmental constraints experienced by families. Adolescents and their parents all discussed the role of work and the importance of earning money to meet basic needs, such as shelter and food. Adolescents in the focus groups often worked to help support their family and stated that they would not attend a programme that interfered with work or with school, for those youth attending school. Adolescents also stated that monsoon season could pose a serious challenge, as the weather could make it too difficult to attend a programme that required them to travel.

Parents were similarly focused on the constraints posed by work and having to meet basic needs associated with daily living. All of the parents had limited economic resources. As one mother stated, "Without work we won't be able to sustain our life." Fathers also noted that their work could necessitate that they travel to other villages or cities and as such, they would not be able to attend a programme that required them to attend multiple sessions. Both mothers and fathers indicated that a programme had to be flexible for their schedules and not interfere with their ability to support their families.

### Recruitment and retention strategies

Adolescent boys and girls provided specific suggestions about how best to recruit and retain them into a family-based programme. Overall, adolescents recommended a face-to-face outreach, conducted by a recruiter who would visit the adolescents' houses to invite them to participate. In addition, adolescents suggested that they would be receptive to hearing from youth already enrolled in a programme, and recommended using village friendship networks as a mechanism to reach large numbers of youth.

For adolescents, successful recruitment efforts would highlight the health benefits of the programme for both youth and the broader community. Both adolescent males and females believed that a family-based programme could have a larger community impact and that this was an important point to publicize.

Mothers and fathers also recommended face-to-face recruitment methods. Overall, parents endorsed a personalized approach, with recruiters going from house to house to provide information on the project. Both mothers and fathers mentioned the importance of drawing upon existing social networks to recruit families and emphasizing how a family-based programme would benefit the future of their children.

Parents also recommended that male recruiters should recruit fathers and sons, and female recruiters should recruit mothers and daughters. For example, one mother stated:

Women from a *pada *should tell people in the same *pada *that a meeting on health is organized and they should come. This information is in the context of the future of our children. If we only don't listen then who will think about the future of our children. All this we can tell in our hamlet.

Similarly, a father recommended an approach where a recruiter could:

.... personally go and speak to them. What do they feel, one must personally try to make them understand and speak. You must tell him that come to the programme if you understand what is being said then make use of it, if not then you can leave the programme.

In addition, fathers felt it was important for recruiters to clearly state the goal of the programme so that families could easily understand its purpose and relevance for their lives.

### Content and format of a family-based intervention

Both adolescent boys and girls wanted accurate, relevant and developmentally appropriate information. Many of the youth in the focus groups stressed the importance of giving "proper advice" about HIV/AIDS. In general, adolescents felt it important to have a proposed family-based intervention that is "comprehensive and includes content both related to abstinence and safer sex". Adolescents expressed interest in knowing both about ways they could avoid becoming sexually active and ways they could protect themselves if they did in fact become sexually active.

Both adolescent boys and girls were clear that a programme had to be flexible, convenient and adolescent friendly. Youth identified a number of characteristics that would make a youth programme friendly, including the use of diverse types of materials and programme activities. Adolescents felt that programme information could be shared through a variety of methods, including skits or plays, songs, and posters, pamphlets and other print materials. Regardless of the medium, adolescents emphasized the importance of addressing illiteracy and suggested that information about a family-based programme needed to be provided orally and in writing, as many of their parents could not read.

Parents wanted current and factual information on HIV/AIDS, strategies for protecting oneself from HIV/AIDS, including correct and consistent condom use, and sexual behaviour. Parents were open to receiving information about HIV/AIDS in a variety of ways, including via written materials and visual images. For written materials, parents stressed the importance of addressing illiteracy in the village and of making materials available in multiple languages, e.g., Hindi and Marathi. As one mother stated, "Now we get paper but we can't even read it ... what you will tell us face to face we will understand from there only." Regardless of the format, both mothers and fathers stressed the importance of making programme materials adolescent friendly.

## Discussion

To date, very few family-based HIV prevention interventions have been developed for rural Indian youth. The majority of interventions have targeted adolescents in schools or health clinics. As a result, a number of questions regarding the feasibility and acceptability of a family-based intervention remain.

To the best of our knowledge, this study is one of the first to conduct focus groups with rural adolescents, mothers and fathers on the feasibility of a comprehensive family-based adolescent HIV prevention intervention. Our findings suggest that a family-based intervention is feasible provided that it: (1) provides families with comprehensive knowledge and strategies about preventing HIV/AIDS; (2) addresses potential barriers to participation; (3) is adolescent friendly, flexible and convenient; and (4) is developmentally and culturally appropriate for rural Indian families.

Overall, both parents and adolescents believed that a family-based programme was feasible and culturally acceptable. Although India is often characterized as having strong cultural barriers to open communication about sex [24], our findings suggest that families are interested in talking with each other about topics like sexual behavior, correct and consistent condom use, and HIV/AIDS. This is an important finding and suggests that family-based approaches are a culturally appropriate and feasible mechanism to help prevent HIV among rural Indian adolescents.

For their part, adolescents respected their parents' opinions, were open to learning about HIV/AIDS from their parents, and identified their parents as important and influential sources of information. At the same time, it is notable that none of the adolescents named their parents as a current source of information or knowledge about HIV/AIDS. This suggests that family communication about HIV/AIDS is low, a finding that has been observed in previous research [6].

In turn, both mothers and fathers believed it was their responsibility to counsel their adolescents on matters related to HIV prevention. Although previous literature has described cultural taboos surrounding the discussion of sexual behaviour in India [8,9], the parents in our study were open and committed to talking with their children. While some participants felt that such discussions could be uncomfortable, previous research with rural Indian families in India has noted that education and training can reduce such discomfort [9].

These findings are important, as they indicate cultural norms and taboos are not immutable, and can be addressed with straightforward intervention activities designed to promote open communication about sensitive topics like HIV/AIDS and sexual behaviour [9].

In addition, programmes will also have to address some parents' fears that talking about HIV/AIDS could have negative consequences for their adolescents. Because the mothers in our study were unable to identify specific negative consequences, additional research is needed to better understand how negative expectancies and other factors influence both parent-adolescent communication about HIV/AIDS and family participation in a family-based HIV prevention programme.

It may be that parents feel they do not have the knowledge to have effective conversations with their children. Indeed, research with families in the US on parent-adolescent communication about sex has identified lack of knowledge as a barrier to communication [52]. Research with Indian families on this topic would be a welcome addition to the literature as it remains underexplored. As a result, it is difficult to make definitive statements about factors at the parental level that may significantly impede or facilitate effective communication about sex and HIV/AIDS.

Theory-based research is necessary to identify the determinants of parent-adolescent communication about sex that can be targeted in the context of a family-based intervention. Such information is necessary if we are to support Indian parents to effectively communicate with their adolescent children about how to reduce their risk of HIV infection.

In addition, research is needed to elucidate the contextual factors associated with increased vulnerability to HIV infection among rural Indian adolescents. One contextual factor that emerged as potentially important was the role of poverty, especially as it relates to youth migration to cities and nearby villages in search of work. A number of researchers have highlighted the complex relationship between poverty and HIV/AIDS [53,54], and there is a need to identify the pathways that underlie this relationship in specific regional contexts.

In our study, poverty appeared to break down the protective role of families when young males were forced to leave home in search of economic opportunities. Mothers believed that this minimized their ability to monitor their children's whereabouts and fathers were concerned about their children's exposure to risk factors, such as commercial sex work. Although none of the parents in our study discussed the relationship between poverty and commercial sex work, other research in India has underscored the role of poverty and economic inequality in young women's entry into sex work [55]. While poverty cannot be ignored as an important contextual factor, HIV prevention interventions targeting HIV risk behaviours must also rely on efficacious methods to prevent or reduce HIV infection.

On a practical level, families provided concrete advice about how best to recruit and retain them in a family-based programme. Parents and adolescents endorsed face-to-face recruitment methods as the most successful way to recruit and retain them in a family-based prevention programme. In addition, parents and adolescents recommended using social networks to outreach to families. This is consistent with previous research, which has identified social networks as an important mechanism to promote communication about sexual health and to inform the design of health prevention programmes in India [9,56].

Parents and adolescents in our study were clear that literacy needs to be addressed. Nationwide, approximately 61% of Indian adults are illiterate [57]. This poses a challenge for delivering information to families where children may have higher rates of literacy than parents. Previous intervention programmes with rural Indian communities have relied on a variety of methods, such as skits, cartoons, pictures and radio programmes, to provide information about HIV/AIDS [9]. Families in our study also endorsed these methods, and future research should explore which mechanism is most appropriate and effective for impacting on behaviour.

Finally, gender emerged as an important consideration, with daughters and fathers voicing support for programmes that fostered same-gender communication in the family system. Numerous studies have observed gender differences in family communication about sex, with mothers communicating more with daughters than with sons [58,59]. Globally, less research has examined father-child communication about sex.

However, recent research with fathers in the US suggests that fathers can be engaged in intervention research focused on adolescent HIV prevention and can be supported to communicate with their sons about topics like sexual behaviour, condoms and HIV [60]. Research on family communication about sex in India is scarce and future studies are needed to more fully understand the nature and extent of such communication, including the role of gender and its potential influence on communication and the development of family-based HIV prevention programmes.

## Conclusion

The findings of the study should be interpreted in the context of the study limitations. First, the study focused on Indian adolescents and their families living in a rural hamlet of Aghai. We did not interview urban families, and the community from which we sampled families was relatively poor. India is a diverse country and our sample may not be representative of other geographical communities. Our study was qualitative in nature; consequently, no causal inferences can be made.

Although demand characteristics (such as taboos against open discussions of sex, HIV/AIDS-related stigma, gender norms for females, and the psychology of group processes) could have influenced participant responses, these potential biases were addressed in several ways.

First, we selected a homogenous sample from a small number of hamlets and separated the groups by gender and generation. Familiarity can impede openness, but it can also promote trust and self-disclosure, as well as enhance participants' comfort in challenging one another. Second, informed consent was obtained from all participants, and the focus groups were conducted in ways to protect participant comfort and confidentiality. Third, focus group moderators were carefully selected and trained. All facilitators received extensive training on how to moderate focus groups, manage group dynamics, and facilitate discussions about sensitive topics like sexual behaviour and HIV/AIDS.

Because of the focus group setting, we did not ask in-depth questions about parent-adolescent communication about sex. As a result, we cannot make definitive statements about the nature of family communication. Future research should explore this topic in both individual in-depth interviews and in survey research with adolescents, mothers and fathers. Here, multiple perspectives will be especially important as they can be used to explore congruency in family reports of parent-adolescent communication about HIV/AIDS and to identify behavioural targets at both the parent and adolescent levels.

Despite these limitations, a strength of this research was the integration of perspectives from adolescent females, adolescent males, mothers and fathers. HIV/AIDS is a disease that affects all members of the family, and research focused on helping Indian adolescents avoid HIV needs to reflect the perspectives of all members of the family system.

In addition, the scope and impact of HIV/AIDS in India necessitates international collaborations that can address the diversity of the epidemic. This study was a collaboration between social scientists in India that was funded by the Indo-US bilateral agreement. It is the first of several formative studies focused on developing an empirical body of literature on how to develop efficacious family-based HIV-prevention programmes for rural Indian youth, and the findings have important implications for researchers interested in developing family-based HIV prevention interventions for Indian adolescents.

## Competing interests

The authors declare that they have no competing interests.

## Authors' contributions

Drs Soletti and Guilamo-Ramos, as principal investigators, had full access to all of the data in the study and take responsibility for the integrity of the data and the accuracy of the data analysis. The study was designed by Drs Guilamo-Ramos, Soletti, Burnette and Bouris. Drs Soletti, Guilamo-Ramos and Burnette and Ms Sharma were responsible for acquiring the data. All authors are responsible for data analysis, interpretation of data, writing of the manuscript, and for the decision to submit the manuscript for publication.
